# Bio-Inspired Soft Robotics: Design, Fabrication and Applications

**DOI:** 10.3390/biomimetics10070447

**Published:** 2025-07-07

**Authors:** Yong Zhong, Pei Jiang, Yi Sun

**Affiliations:** 1 Shien-Ming Wu School of Intelligent Engineering, South China University of Technology, Guangzhou 510640, China; 2 The State Key Laboratory of Mechanical Transmission, Chongqing University, Chongqing 400044, China; peijiang@cqu.edu.cn; 3 Department of Mechano-Informatics, University of Tokyo, Tokyo 113-8656, Japan; y-sun@isi.imi.i.u-tokyo.ac.jp

The field of soft robotics has witnessed a remarkable surge in interest and innovation over the past decade, with bio-inspiration playing a pivotal role in driving forward the design, fabrication, and applications of these adaptable and resilient robotic systems. This book, titled “Bio-Inspired Soft Robotics: Design, Fabrication and Applications,” compiles ten cutting-edge articles that showcase the latest advancements and diverse applications in this burgeoning field. These contributions highlight the multifaceted ways in which nature’s ingenuity can be harnessed to create soft robots that are not only highly functional but also capable of operating in complex and dynamic environments.

One of the key themes explored in this book is the design of soft robots inspired by the unique locomotion strategies of various animals. For instance, Yuqiao Dai et al. [Contribution 1] present a novel approach for controlling flexible continuum robots, leveraging neural networks to model the relationship between motor inputs and shape outputs. This work underscores the potential of bio-inspired design principles in enhancing the precision and adaptability of soft robotic systems. Similarly, Emmanouil Papadakis et al. [Contribution 2] introduce a soft robotic arm that mimics the muscular structure and movement capabilities of an octopus arm. The authors’ detailed design process and experimental evaluation demonstrate the arm’s ability to perform complex tasks such as bending, elongation, and twisting, thereby expanding the scope of potential applications for soft robotic arms.

The fabrication techniques for bio-inspired soft robots are another focal point of this book. Xiaohui Wang et al. [Contribution 3] describe the development of a soft robotic glove for hand rehabilitation, featuring bidirectional bending actuators and a programmable pneumatic control platform. This work exemplifies how innovative fabrication methods can be employed to create soft robotic devices that are both functional and user-friendly. Additionally, Qian Yang et al. [Contribution 4] explore the design and testing of a high-frequency wire-driven robotic fish, highlighting the importance of optimizing the mechanical design and control strategies to achieve efficient and agile underwater locomotion.

The applications of bio-inspired soft robots span a wide range of fields, ranging from medical rehabilitation to environmental exploration. Yihan Wang et al. [Contribution 5] investigate the hydrodynamic performance of a quadrupedal paddling model, providing valuable insights into the design of amphibious robots that can navigate both land and water environments. This research not only advances our understanding of bio-inspired locomotion but also paves the way for the development of versatile robots capable of performing diverse tasks in challenging terrain. Furthermore, Deli Xia et al. [Contribution 6] present a 3D-printed soft robot that mimics the movement of an inchworm, showcasing the potential of combining advanced fabrication technologies with bio-inspired design to create robots that can adapt to complex environments and perform tasks such as obstacle climbing and surface transition. Huibin Liu et al. [Contribution 7] present a quadruped soft robot driven by magnetic fields. The robot is designed to mimic the movement patterns of quadrupeds and can perform crawling and tumbling motions. This multimodal motion capability enables the robot to navigate complex environments, cross obstacles, and deliver cargo in a targeted manner, demonstrating the potential of soft robots in biomedical applications such as targeted drug delivery.

In addition to these notable contributions, the book features several other articles that delve into various aspects of bio-inspired soft robotics. These include studies on the development of novel actuators, sensors, and control systems [Contribution 8], [Contribution 9], as well as explorations of the potential applications of soft robots in fields such as healthcare, environmental monitoring, and search and rescue operations [Contribution 10]. Collectively, these articles provide a comprehensive overview of the current state of the art in bio-inspired soft robotics and highlight the vast potential of this field for future research and development.

As we compile these ten articles into a book, it is evident that the field of bio-inspired soft robotics is at an exciting juncture, with researchers continually pushing the boundaries of what is possible. The innovative designs, fabrication techniques, and applications showcased in this book serve as a testament to the creativity and ingenuity of the scientific community working in this area. We hope that this collection of articles will inspire further research and development, fostering the growth of bio-inspired soft robotics and its applications in various domains. The potential for these robots to revolutionize industries, improve quality of life, and contribute to our understanding of the natural world is immense, and we look forward to witnessing the continued evolution and impact of this fascinating field.

We would like to express our sincere gratitude to all the authors who contributed their valuable research to this book, as well as to the reviewers who provided their expertise and constructive feedback. Their efforts have made this book a rich and informative resource for the soft robotics community. We also extend our appreciation to the editorial team at MDPI for their support and dedication in bringing this Special Issue to fruition.

